# Prevalence and Factors Associated with Anaemia Among Sub-Saharan African Adolescents: A Systematic Review and Meta-Analysis of Nutritional, Socio-Demographic, Environmental, and Infectious Factors

**DOI:** 10.3390/nu18152404

**Published:** 2026-07-23

**Authors:** Sisa H. Martins, Mamakase G. Sello, Musawenkosi Ndlovu, Marakiya T. Moetlediwa, Samukelisiwe S. Madlala, Joel Choshi, Phiwayinkosi Dludla, André P. Kengne, Zandile J. Mchiza, Sihle E. Mabhida

**Affiliations:** 1 Non-Communicable Diseases Research Unit, South African Medical Research Council, Tygerberg 7505, South Africa or 4528499@myuwc.ac.za (S.H.M.); marakiya.moetlediwa@mrc.ac.za (M.T.M.); samukelisiwe.madlala@mrc.ac.za (S.S.M.); andre.kengne@mrc.ac.za (A.P.K.);; 2 School of Public Health, University of the Western Cape, Bellville 7535, South Africa; desmasello@gmail.com; 3 Department of Nutrition, Faculty of Health Sciences National University of Lesotho, Maseru 100, Lesotho; 4 Division of Pharmaceutical Chemistry, Faculty of Pharmacy, Rhodes University, Makhanda 6140, South Africa; muser165@gmail.com; 5 Department of Human Biology, Faculty of Health Sciences, University of Cape Town, Observatory 7925, South Africa; 6 Department of Physiology and Environmental Health, University of Limpopo, Sovenga 0727, South Africa; joel.choshi@ul.ac.za; 7 Department of Biochemistry and Microbiology, University of Zululand, KwaDlangezwa 3886, South Africa; dludlap@unizulu.ac.za; 8 School of Medicine, University of KwaZulu-Natal, Durban 4000, South Africa; 9 Department of Medicine, University of Cape Town, Cape Town 7700, South Africa; 10 African Population and Health Research Center, Nairobi P.O. Box 10787-00100, Kenya; 11 Health Systems Research Unit, South African Medical Research Council, Cape Town 7505, South Africa

**Keywords:** anaemia, adolescents, Sub-Saharan Africa, prevalence, systematic review, meta-analysis, nutritional determinants, socio-demographic factors

## Abstract

**Background/Objectives**: Anaemia is a critical public health challenge in Sub-Saharan Africa (SSA), particularly among adolescents. The objective was to estimate the pooled prevalence of anaemia among adolescents in SSA and synthesise evidence on its nutritional and contextual determinants. **Methods**: A systematic search of databases including PubMed, CINAHL and Web of Science was conducted for studies published from January 2000 to October 2025. The methodological approach was guided by the Preferred Reporting Items for Systematic Reviews and Meta-Analyses (PRISMA) guidelines. Pooled prevalence estimates were computed using random-effects models, and heterogeneity was assessed using the *I*^2^ statistic and Cochran’s Q test. Subgroup and meta-regression analyses were performed to explore sources of variation. Determinants were synthesised as pooled odds ratios with 95% confidence intervals. Publication bias was evaluated through funnel plot asymmetry and Egger’s regression test. **Results**: Forty-eight (*n* = 48) studies involving 76,596 adolescents met the inclusion criteria. The pooled prevalence of anaemia was 33% (95% CI: 0.28–0.39), with substantial heterogeneity across studies. Factors associated with higher odds of anaemia included low dietary diversity (OR 1.74, 95% CI: 1.10–2.74) and poor iron or micronutrient supplementation adherence or lack of supplementation (OR 1.94, 95% CI: 1.25–3.01). Infection-related exposures (OR 3.03, 95% CI: 1.99–4.61) and environmental determinants (OR 2.23, 95% CI: 1.41–3.55) were also significantly associated with anaemia. Socio-demographic factors associated with anaemia included menstrual factors among girls (OR 2.51, 95% CI: 1.58–3.99), household size (OR 3.32, 95% CI: 1.61–6.85), awareness (OR 1.71, 95% CI: 1.08–2.70), and age group (OR 2.37, 95% CI: 1.47–3.83). **Conclusions**: Anaemia among adolescents in SSA remains an important public health concern, although the pooled estimates should be interpreted cautiously because of substantial heterogeneity and methodological differences across studies. The findings suggest that adolescent anaemia is associated with multiple nutritional and contextual factors, but more consistent and context-specific research is needed to refine regional estimates and guide interventions.

## 1. Introduction

Anaemia, defined as an insufficient number of healthy red blood cells or low haemoglobin concentration, reduces the blood’s capacity to transport oxygen to body tissues and organs. This condition remains a major public health concern among adolescents worldwide, with the greatest burden reported in low- and middle-income regions, particularly Sub-Saharan Africa (SSA) [1]. Recent estimates suggest that the prevalence of adolescent anaemia ranges from 35.7% to 47.4% in some parts of SSA, including Central Africa and Western Africa [2,3]. In adolescents, anaemia is commonly classified using age- and sex-specific haemoglobin thresholds recommended by the World Health Organization [4].

Adolescence is a particularly vulnerable period for anaemia because rapid physical growth increases iron requirements. Among girls, this vulnerability is further exacerbated by the onset of menstruation and the risk of early pregnancy [5,6]. Anaemia can impair cognitive development, immune function, school performance, and long-term health outcomes [7,8]. Identifying its burden and key determinants among adolescents is therefore important for guiding context-specific prevention strategies, strengthening adolescent nutrition programmes, and reducing avoidable health and educational inequalities in SSA.

Anaemia in adolescents is multifactorial, arising from a combination of nutritional, infection-related, environmental, and socio-demographic factors [9]. Nutritional contributors to anaemia are particularly important in sub-Saharan Africa, where poor nutrition outcomes remain common in many food-insecure settings. These contributors include poor dietary diversity, inadequate intake of iron-rich foods, poor adherence to iron and folate supplementation, and dietary practices that reduce iron absorption. Together, these factors can compromise haemoglobin synthesis, especially during periods of rapid growth [10,11,12]. On the other hand, infections such as malaria and intestinal parasites further contribute to anaemia through blood loss, inflammation, and impaired nutrient absorption [13]. In Africa, socio-demographic and environmental disadvantages such as poverty, rural residence, low parental education, and household deprivation can increase anaemia risk by limiting access to nutritious food and healthcare and increasing exposure to infection [2].

Although evidence on adolescent anaemia in Africa is growing, much of the literature remains fragmented. Many studies on anaemia are limited to specific countries or regions, focus mainly on girls, or examine determinants in isolation [5,14,15]. Clinical and infection-focused studies also show that malaria and soil-transmitted helminth co-infections are strongly associated with anaemia, but such evidence is often context-specific and not consistently reflected in broader pooled estimates [16]. Therefore, updated pooled evidence across African settings is needed to clarify the combined determinants of anaemia among adolescents. Consequently, there is limited synthesis of how nutritional, socio-demographic, environmental, and infection-related factors collectively influence anaemia risk among adolescents across the region [5,9,17]. To address this gap, this systematic review and meta-analysis examined the prevalence of anaemia among adolescents in SSA and synthesised evidence on its nutritional, socio-demographic, environmental, and infection-related determinants.

## 2. Materials and Methods

### 2.1. Study Design

This systematic review included studies published between January 2000 and October 2025 to examine the prevalence and determinants of anaemia among adolescents aged 10–19 years in SSA, with a specific focus on nutritional, socio-demographic, environmental, and infection-related factors. The primary objective was to estimate the pooled prevalence of anaemia in adolescents from SSA. The secondary objective was to synthesize evidence on its key nutritional and contextual determinants within the analyzed population. The review was conducted in accordance with the Preferred Reporting Items for Systematic Reviews and Meta-Analyses (PRISMA) guidelines. The study protocol was registered in PROSPERO (International Prospective Register of Systematic Reviews) under the identifier CRD420251117146.

### 2.2. Information Sources and Search Strategy

The main electronic databases and search engines that were explored to identify relevant literature included PubMed (*n* = 524), CINAHL (*n* = 493) and Web of Science (*n* = 489). The Google Scholar search engine was used to find additional studies. For Google Scholar, search results were sorted using the platform’s default relevance ranking, and the first *n* = 200 titles and abstracts were screened. This cutoff was selected as a pragmatic balance between sensitivity and feasibility because relevance typically declines markedly beyond the first several pages of results, while the highest-ranked records are most likely to include eligible studies. Additional articles were identified through manual searches, including screening of the reference lists of selected papers. The final literature search was conducted on 31 October 2025 for Pub-Med, CINAHL, Web of Science, and Google Scholar. Reference list screening was completed immediately thereafter.

The search strategy incorporated keywords and MeSH terms relating to the target population (adolescent* OR teen* OR youth OR “secondary school”), the outcome of interest (anemia OR anaemia OR iron deficiency OR haemoglobin OR hemoglobin; MeSH terms including “anaemia” and “anemia, iron-deficiency”), and geographic identifiers for sub-Saharan Africa. The geographic component was applied using SSA-related terms, names of individual SSA countries, and regional terms such as “Africa South of the Sahara,” “Africa, Eastern,” and “Africa, Western,” rather than relying only on a broad Africa filter. Exposures such as nutrition, nutritional status, diet, malnutrition, micronutrients, iron, socio-demographic, socioeconomic, education, income, and factors associated with anaemia (including MeSH terms “Prevalence”, “Epidemiology”, and “Determinants”) were also included. Boolean operators were applied to these terms to capture all relevant studies examining anaemia and its nutritional and socio-demographic determinants among adolescents across SSA. The full search strategy is available in Supplementary File S1.

### 2.3. Eligibility Criteria and Study Selection

Study eligibility was defined using the PEO framework: Population, Exposure, and Outcome. The population of interest comprised adolescents aged 10–19 years residing in countries located in SSA. The exposures of interest included nutritional and socio-demographic factors, such as dietary intake, nutritional status, micronutrient status, malnutrition, socioeconomic status, educational attainment, income, and other relevant social or demographic characteristics. The outcomes of interest were the prevalence of anaemia, iron deficiency anaemia and haemoglobin concentration among adolescents. Eligible study designs included cross-sectional, cohort, and case–control studies. Randomised controlled trials were also considered eligible when they provided baseline prevalence data or observationally relevant determinant data for adolescents.

Studies were included if they were published in English between January 2000 and October 2025 and reported data on adolescents aged 10–19 years in SSA. The 2000–2025 period was selected to capture contemporary evidence generated during the Millennium Development Goals and Sustainable Development Goals eras, when anaemia prevention, adolescent nutrition, malaria control, deworming, and food security interventions became major public health priorities in the region [13,18]. The age range of 10–19 years was used because it follows the World Health Organization’s standard definition of adolescence [19]. We excluded studies if they did not report the prevalence of anaemia or IDA, did not provide haemoglobin concentration data, or did not examine determinants of anaemia relevant to the review objectives. Reviews, conference abstracts, editorials, commentaries, non-peer-reviewed publications, interventional studies without relevant baseline data, and articles published in languages other than English were excluded.

**Figure 1:**
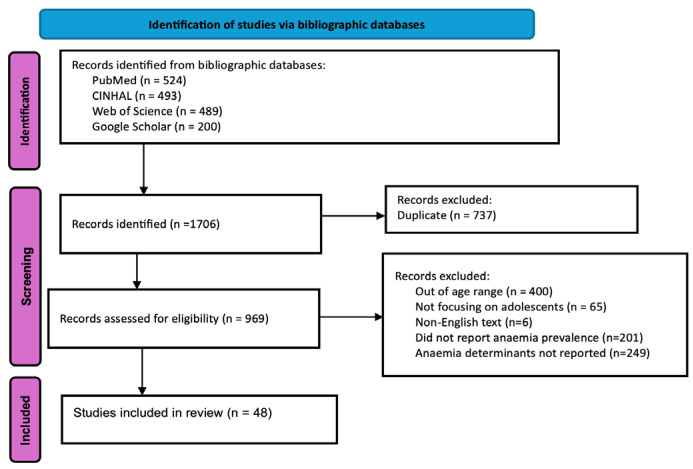
A PRISMA flow diagram presenting the study selection and inclusion process of studies.

The eligibility screening process followed two stages. First, titles and abstracts were screened based on the records identified through electronic database searches. Second, articles that appeared relevant or lacked sufficient information for a clear decision were retrieved and assessed in full text. All references were exported into EndNote (Clarivate, Philadelphia, PA, USA) for de-duplication and then uploaded into Rayyan (Rayyan Systems Inc., Cambridge, MA, USA) for screening. Two reviewers (MGS and SHM) independently screened titles and abstracts and subsequently assessed the full texts of potentially eligible articles. Any discrepancies were resolved through discussion or adjudication by a third reviewer (SEM). The PRISMA diagram summarising the study selection process is presented in Figure 1.

### 2.4. Data Extraction

Two reviewers (SHM and MGS) independently retrieved data from the included studies using a standardized extraction procedure documented in a Microsoft Excel spreadsheet (Microsoft Corporation, Redmond, WA, USA). Reviewers independently extracted the study characteristics including author, year, country, design, setting, sample size and participant characteristics. Definitions and measurement methods for anaemia; prevalence estimates; and nutritional determinants as well as the socio-demographic determinants were extracted. Any disagreements were resolved by consensus or arbitration. Anaemia definitions were extracted as reported in each primary study. Where studies applied WHO criteria, anaemia was generally defined as haemoglobin < 11.5 g/dL for adolescents aged 10–11 years, <12.0 g/dL for adolescents aged 12–14 years and non-pregnant girls aged 15–19 years, and <13.0 g/dL for boys aged 15–19 years. For pregnant adolescents, altitude-adjusted populations, and studies reporting iron deficiency anaemia, study-specific thresholds were extracted as reported and considered during interpretation.

### 2.5. Risk of Bias and Quality Assessment

Two reviewers [MGS and SHM] independently appraised the methodological quality of the included studies using the Joanna Briggs Institute’s (JBI) critical appraisal checklists for studies reporting prevalence data [20]. This checklist is one of the JBI critical appraisal tools developed to support assessment of methodological quality and risk of bias across different study designs, including prevalence and observational studies [21]. The checklist assesses the appropriateness of the sample frame, sampling procedure, sample size, description of study subjects and setting, coverage of data analysis, validity and reliability of outcome measurement, appropriateness of statistical analysis, and response rate management. Disagreements between the two reviewers were resolved through discussion or consultation with a third reviewer [SEM] where necessary.

### 2.6. Data Synthesis

Prevalence and determinant syntheses were treated as related but distinct review objectives. Meta-analysis was undertaken only when at least three studies reported sufficiently comparable outcomes and exposures. For determinant analyses, adjusted effect estimates were preferentially extracted where available, and pooled findings were interpreted cautiously when definitions and adjustment set varied across studies. Pooled effect sizes (e.g., odds ratios, prevalence estimates) were calculated with 95% confidence intervals (CIs) using a random-effects model (DerSimonian and Laird method). The Freeman–Tukey double arcsine transformation was applied to stabilise variances of raw proportions prior to pooling. Statistical heterogeneity among studies was assessed using the *I*^2^ statistic and τ^2^, which quantify the proportion of total variation and the between-study variance, respectively. To explore potential sources of between-study heterogeneity, mixed-effects meta-regression analyses were performed using the restricted maximum likelihood (REML) estimator. Study-level covariates were selected a priori based on their potential influence on anaemia prevalence estimates and included study setting (mixed, urban, rural, and refugee camps), publication year, sample size, and haemoglobin assessment method. Haemoglobin assessment methods were categorized as laboratory-based cyanmethemoglobin methods, HemoCue point-of-care devices, other portable haemoglobinometers, and unclear/unspecified methods. Publication bias was evaluated through funnel plot asymmetry and Egger’s regression test. Study quality and risk of bias assessments are integrated into the interpretation of findings. Where exposure definitions differed across studies, determinants were pooled only when they were judged to be conceptually comparable; otherwise, findings were synthesized narratively.

## 3. Results

### 3.1. Characteristics of Included Studies

Figure 1 provides an overview of the study selection process. Briefly, a total of 1706 records were identified through database searches and other sources. After removing 737 duplicates, 969 titles and abstracts were screened, resulting in 112 studies selected for full-text review. Of these, 48 articles met the inclusion criteria and were encompassed as part of the final analysis. Study characteristics are summarized in Table 1.

Altogether, the studies included 76,596 participants, with individual study sample sizes ranging from 120 to 39,712. Across all studies, there were more females (*n* = 66,734) than males (*n* = 9862). Most of the included studies were conducted in Ethiopia (*n* = 21) and Ghana (*n* = 8), while the remaining studies were from Tanzania (*n* = 3), Sudan (*n* = 2), Kenya (*n* = 2), Burkina Faso (*n* = 2), Uganda (*n* = 2), Nigeria (*n* = 2), Cameroon (*n* = 1), South Africa (*n* = 1), and Benin (*n* = 1), with three multicountry studies (Ethiopia, Sudan, Tanzania (*n* = 1); 33 Sub-Saharan African countries; (*n* = 1) and Kenya and Nepal (*n* = 1)). Forty-six studies were cross-sectional study designs, while two were randomized controlled trials. The concentration of studies from Ethiopia and Ghana may have disproportionately influenced the pooled prevalence estimate and may limit the representativeness of the pooled estimate for under-studied SSA settings.

### 3.2. Risk of Bias and Quality Assessment

The risk of bias and methodological quality were assessed for 48 included studies using the JBI critical appraisal checklist for prevalence studies. The methodological quality of the included studies was moderate to high. Thirty-three studies were rated as suitable for inclusion, while 15 were included with caution due to methodological limitations (Supplementary File S2). Most studies performed well across the JBI domains. Twenty-four studies met all nine criteria, nine met eight, and seven met seven. All studies adequately described the study subjects and setting. Forty-seven studies used appropriate statistical analyses, 44 used valid methods for identifying anaemia, and 45 measured the condition in a standard and reliable way. The main sources of potential bias were related to sampling, sample size reporting, data coverage, and response rate management. Eight studies had unclear sampling procedures, and one study used an inappropriate sampling approach. Similarly, eight studies did not clearly justify sample size adequacy, while one study was judged to have an inadequate sample size. Ten studies had unclear data coverage. The weakest domain was response rate reporting. Only 29 studies clearly reported an adequate response rate or appropriate management of non-response; 16 were unclear; and 3 were not applicable. The assessment indicates that most studies were methodologically acceptable for estimating anaemia prevalence among adolescents. However, findings should be interpreted with consideration of recurring concerns related to representativeness, unclear sampling procedures, limited reporting of response rates, and the inclusion of some studies rated as include with caution.

### 3.3. Pooled Prevalence of Anaemia

**Figure 2:**
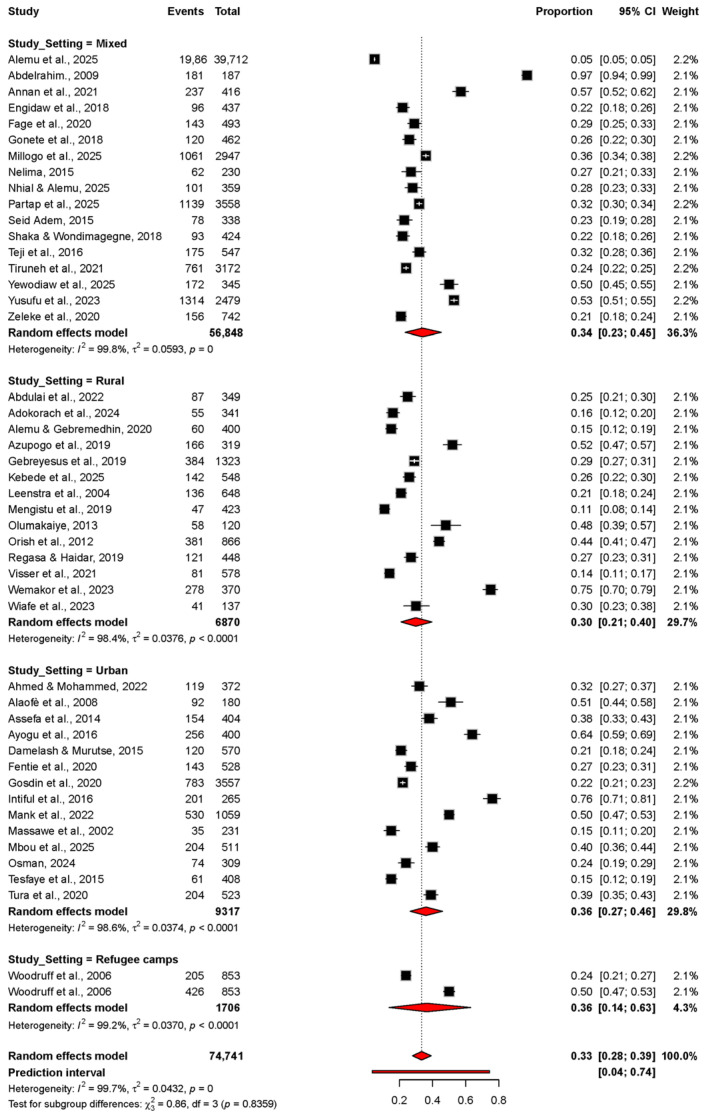
Forest plot of pooled prevalence of anaemia among adolescents in SSA by study setting. Subgroups include mixed, urban, rural, and refugee camp settings. Squares represent study-specific prevalence estimates, with square size proportional to study weight; horizontal lines indicate 95% confidence intervals; diamonds represent pooled random-effects estimates.

A total of 47 studies involving 74,741 adolescents reported data on anaemia prevalence and were included in the pooled prevalence analysis. The included studies showed considerable variation in anaemia prevalence across settings, suggesting important contextual differences in population characteristics, study design, and exposure profiles. The random-effects meta-analysis showed that the pooled prevalence of anaemia among adolescents in SSA was 33% (95% CI: 0.28–0.39) (Figure 2); however, substantial heterogeneity indicates considerable variations across study settings and populations, limiting interpretation as a precise estimate.

In subgroup analyses by study setting, the pooled prevalence was 34% (95% CI: 0.23–0.45) in mixed-setting studies, 36% (95% CI: 0.27–0.46) in urban studies, 30% (95% CI: 0.21–0.40) in rural studies, and 36% (95% CI: 0.14–0.63) in refugee camp studies. Heterogeneity remained extremely high within each subgroup, indicating substantial variability between studies that was not explained by study setting alone. The test for subgroup differences was statistically significant (χ^2^ = 17.53, df = 4, *p* = 0.0054), indicating that anaemia prevalence differed by study setting.

### 3.4. Nutritional Associated Factors

**Figure 3:**
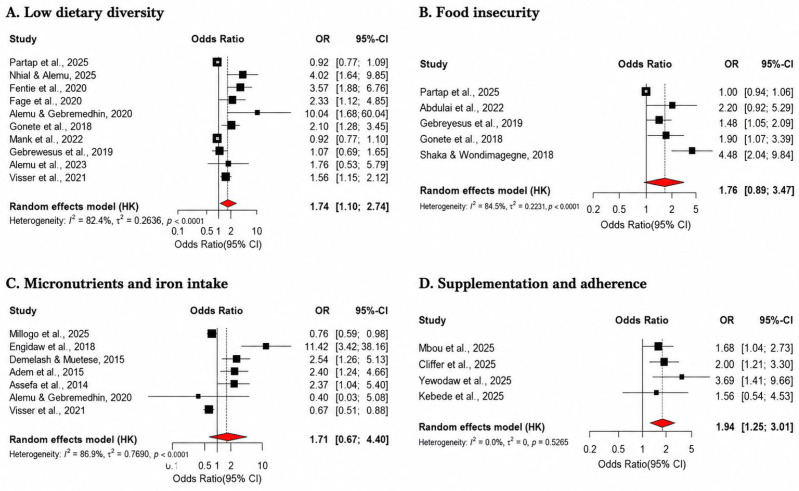
Forest plots of pooled nutritional factors associated with anaemia among adolescents in SSA. Panel (**A**) shows low dietary diversity, Panel (**B**) food insecurity, Panel (**C**) micronutrients and iron intake, and Panel (**D**) supplementation and adherence. Odds ratios greater than 1 indicate higher odds of anaemia in the exposed group. Squares represent study-specific odds ratios, with square size proportional to study weight; horizontal lines indicate 95% confidence intervals; diamonds represent pooled random-effects estimates.

The pooled analyses of nutritional associated factors showed that low dietary diversity was significantly associated with increased odds of anaemia (OR 1.74, 95% CI: 1.10–2.74). However, substantial heterogeneity was observed across the included studies (*I*^2^ = 82.4%) (Figure 3), indicating that the pooled odds ratios should be interpreted as the summary of associations rather than independent causal effects. In contrast, food insecurity was not significantly associated with anaemia in the pooled analysis (OR 1.76, 95% CI: 0.89–3.47), with high heterogeneity (*I*^2^ = 84.5%). For micronutrients and iron intake, the pooled association was elevated but not statistically significant (OR 1.71, 95% CI: 0.67–4.40), with substantial heterogeneity (*I*^2^ = 86.9%). In contrast, poor supplementation adherence or lack of supplementation showed a statistically significant association with anaemia (OR 1.94, 95% CI: 1.25–3.01) with no observed heterogeneity (*I*^2^ = 0.0%). This finding should be interpreted cautiously because food insecurity measures varied across studies and may not adequately capture dietary quality, micronutrient intake, or intra-household food distribution.

### 3.5. Infection-Related and Environmental Determinants

**Figure 4:**
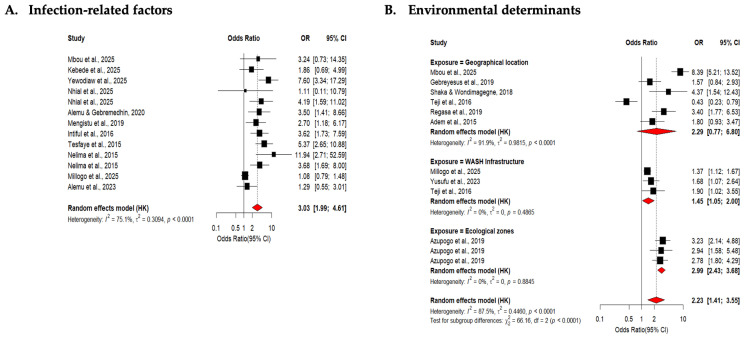
Forest plots of pooled infection-related and environmental factors associated with anaemia among adolescents in SSA. Panel (**A**) shows infection-related factors, while Panel (**B**) shows environmental determinants including geographical location, WASH infrastructure, and ecological zone subgroups. Odds ratios greater than 1 indicate higher odds of anaemia.

Infection-related exposures were significantly associated with anaemia (OR = 3.03, 95% CI: 1.99–4.61), although substantial heterogeneity was observed across studies (*I*^2^ = 75.1%) (Figure 4). Environmental determinants were also associated with anaemia overall (OR = 2.23, 95% CI: 1.41–3.55), with high heterogeneity (*I*^2^ = 87.5%) and significant subgroup differences. Within this domain, WASH-related factors showed a significant association (OR = 1.45, 95% CI: 1.05–2.00; *I*^2^ = 0%), while ecological zones also showed a significant association (OR = 2.99, 95% CI: 2.43–3.68; *I*^2^ = 0%). In contrast, geographical location was positively associated with anaemia but was not statistically significant (OR = 2.29, 95% CI: 0.77–6.80), with very high heterogeneity (*I*^2^ = 91.9%).

### 3.6. Socio-Demographic Determinants

**Figure 5:**
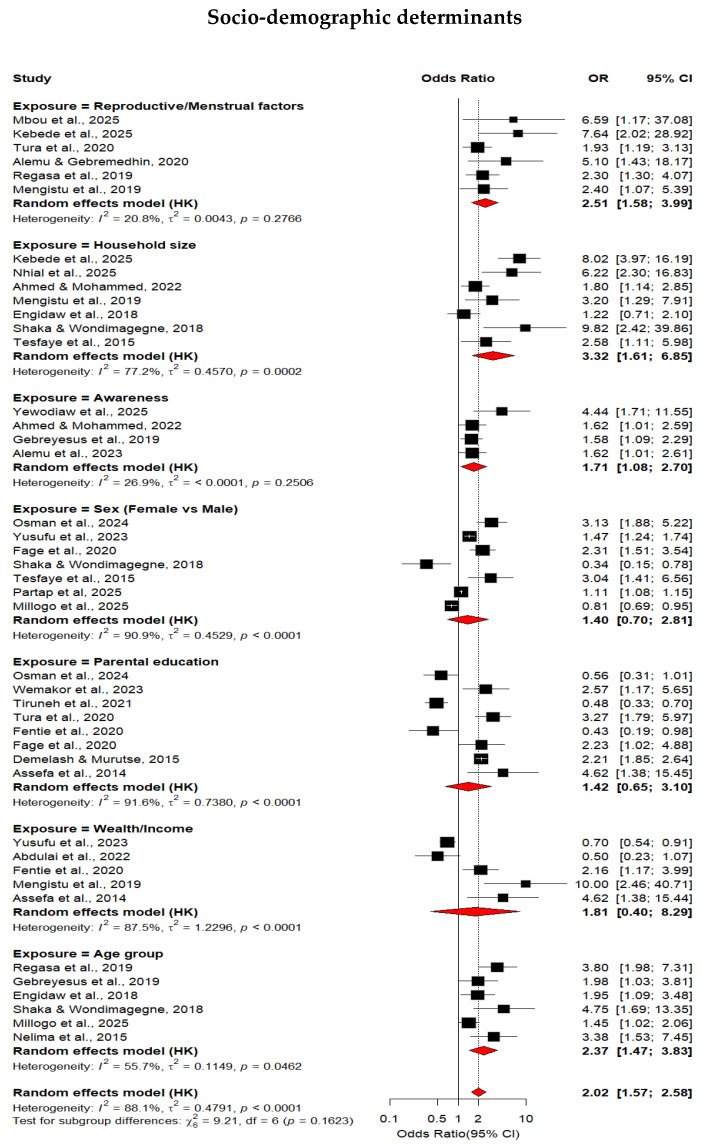
Forest plot of pooled socio-demographic factors associated with anaemia among adolescents in SSA, including reproductive or menstrual factors, household size, awareness, sex, parental education, wealth or income, and age group. Odds ratios greater than 1 indicate higher odds of anaemia in the exposed group. Squares represent study-specific odds ratios, with square size proportional to study weight; horizontal lines indicate 95% confidence intervals; diamonds represent pooled random-effects estimates.

The pooled socio-demographic analyses showed significant associations for reproductive or menstrual factors (OR 2.51, 95% CI: 1.58–3.99), household size (OR 3.32, 95% CI: 1.61–6.85), awareness (OR 1.71, 95% CI: 1.08–2.70), and age group (OR 2.37, 95% CI: 1.47–3.83) (Figure 5). On the other hand, pooled effects for sex (OR 1.40, 95% CI: 0.70–2.81), parental education (OR 1.42, 95% CI: 0.65–3.10), and wealth/income (OR 1.81, 95% CI: 0.40–8.29) were statistically insignificant. When all socio-demographic subgroup estimates were pooled together, the overall summary effect remained statistically significant (OR = 2.02, 95% CI: 1.57–2.58).

### 3.7. Meta-Regression

The overall model was not statistically significant (QM = 9.65, df = 9, *p* = 0.380), indicating that the included moderators did not collectively explain a significant proportion of the between-study heterogeneity (Table 2). Study setting, publication year, and haemoglobin assessment method were not significantly associated with anaemia prevalence estimates (all *p* > 0.05). Sample size demonstrated a small but statistically significant inverse association with prevalence estimates (β = −0.0001, *p* = 0.008). Despite inclusion of these moderators, substantial residual heterogeneity remained (*I*^2^ = 99.08%; *p* < 0.001), suggesting that other unmeasured factors contributed to the observed variability across studies.

In the determinant analyses, sample size was significantly associated with heterogeneity across several domains, including socio-demographic factors, dietary diversity, anthropometric undernutrition, infection-related factors, and environmental determinants. The publication year was only significant for environmental determinants. The full determinant meta-regression findings are provided in (Appendix A).

### 3.8. Publication Bias

**Figure 6:**
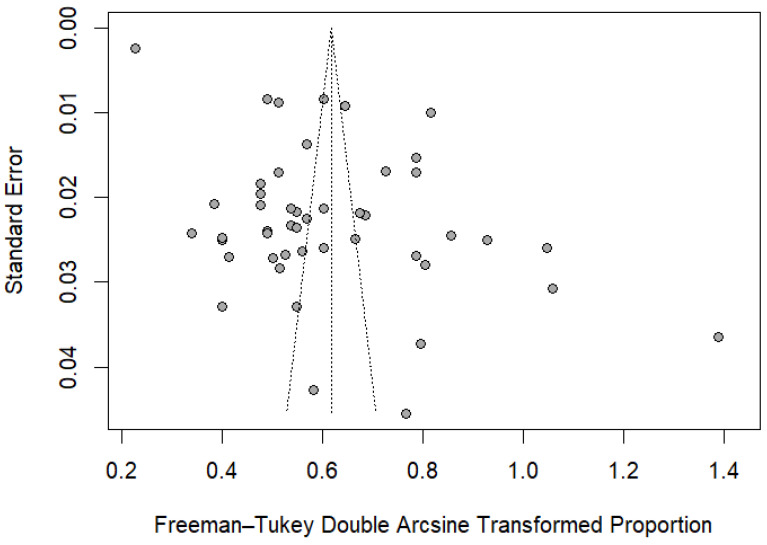
Funnel plot showing the distribution of studies around the pooled estimate suggests some asymmetry, indicating possible small-study effects or publication bias.

Visual inspection of the funnel plot suggested some degree of asymmetry, indicating possible small-study effects and/or publication bias (Figure 6). However, this should be interpreted cautiously because the pooled prevalence analysis was characterized by very high heterogeneity and unequal geographic representation, and funnel plot asymmetry under such conditions may reflect between-study differences rather than publication bias alone.

## 4. Discussion

The current review provides a pooled estimate of the burden of anaemia among adolescents in SSA, showing that approximately one in three adolescents is affected. This estimate was based on 47 studies involving 74,741 participants and yielded a pooled prevalence of 33%. Interestingly, this burden was observed across rural, urban, and mixed settings, with similarly high estimates in urban and mixed populations, suggesting that adolescent anaemia is not confined to rural areas. Overall, the findings suggest that anaemia among adolescents in SSA is widespread and multifactorial, with low dietary diversity, poor iron or micronutrient supplementation adherence, infection-related exposures, environmental conditions, menstrual factors, large household size, low anaemia awareness, and age group associated with higher odds across different local contexts.

Among the nutritional factors examined, low dietary diversity was associated with higher odds of anaemia. However, the substantial heterogeneity across studies limits the strength of this interpretation. This finding is nevertheless consistent with regional evidence linking poor diet quality and inadequate intake of nutrient-dense foods to anaemia and related nutritional deficiencies [17,69]. This is particularly important during adolescence, when rapid growth increases nutrient requirements; however many adolescents may adopt poor dietary practices such as meal skipping, frequent snacking, low meal frequency, and limited consumption of iron-rich foods. These behaviours can reduce intake of essential micronutrients needed for haemoglobin synthesis and may partly explain the observed association between poor dietary diversity and anaemia [70]. In contrast, food insecurity was not significantly associated with anaemia in the pooled analysis, and this finding should be interpreted cautiously given the possibility of limited statistical power and high between-study heterogeneity. Food insecurity measures varied across studies and may not adequately capture dietary quality, micronutrient intake, or intra-household food distribution.

This nutritional pattern is further supported by the more consistent pooled association observed for supplementation and adherence. However, this finding should still be interpreted cautiously, considering the number, design, and context of the contributing studies. This interpretation is consistent with recent evidence indicating that weekly iron–folic acid supplementation can reduce anaemia risk in adolescent girls, while poor adherence remains a key barrier to programme effectiveness [28,71,72]. At the same time, the imprecise pooled estimate for micronutrient and iron intake may largely reflect limited statistical power, especially where few studies contributed data, with additional variability arising from differences in dietary assessment methods and definitions of nutrient adequacy [73,74,75].

Beyond nutrition, the significant pooled association for infection-related factors highlights that adolescent anaemia in SSA cannot be conceptualised as a nutrition-only problem. This finding is consistent with recent evidence showing that parasitic infection, recent illness, and related infectious exposures were associated with anaemia in adolescents, often occurring alongside poor nutritional status [14,17,28]. Poor Hb status among adolescents in resource-limited, high-infection settings may reflect multiple biological mechanisms, including inflammation, blood loss, hemolysis, and reduced nutrient absorption, which together reinforce the interaction between infectious and nutritional pathways. Similarly, the pooled environmental analyses suggest that broader living conditions appear relevant to anaemia risk. This is supported by recent evidence indicating that hygiene-related factors, sanitation, and water access are linked to anaemia and related nutritional outcomes, while ecological and place-based differences may shape exposure to infection, food availability, and access to services [14,76,77]. Although the environmental category remained highly heterogeneous, the overall pattern supports the view that structural environmental conditions matter, even if their effects vary across settings that differ in sanitation infrastructure, water access, and ecological conditions.

The socio-demographic pooled analyses further support interpreting adolescent anaemia through a broader social determinants lens. Significant associations for menstrual or reproductive factors, household size, awareness, and age suggest that physiological demand, household resource constraints, knowledge barriers, and developmental stage all influence vulnerability. This interpretation is consistent with recent evidence from SSA showing that adolescent anaemia and related nutritional disadvantages are socially patterned and shaped by reproductive status, poverty, education, and household conditions [22,24].

Taken together, these findings have important implications for policy, practice, and future research in SSA. Adolescent anaemia should not be viewed as an isolated nutritional disorder, but as a multifactorial condition shaped by overlapping nutritional, infectious, environmental, and socio-demographic factors. This supports the need for integrated, context-specific interventions that go beyond iron supplementation alone and combine dietary improvement, iron or iron–folic acid supplementation, infection prevention and treatment, adolescent health education, and broader efforts to improve water, sanitation, healthcare access, and household living conditions.

### Strengths and Limitations

A key strength of this review is that it goes beyond pooled prevalence to synthesise nutritional, infection-related, environmental, and socio-demographic determinants of adolescent anaemia across SSA, thereby supporting a more holistic interpretation of risk. The subgroup analyses and meta-regression also add value by showing that variability is driven more by differences in study settings and populations than by simple time trends. However, several limitations should be considered when interpreting these findings. First, heterogeneity was substantial across prevalence and determinant analyses, reflecting differences in study populations, exposure definitions, anaemia thresholds, and measurement methods. Furthermore, studies included varied diagnostic thresholds, participant composition, age distributions, and haemoglobin assessment methods. Although random-effects models account for some between-study variability, methodological differences may limit comparability and contribute to residual heterogeneity.

Second, the concentration of studies from Ethiopia and Ghana may have disproportionately influenced the pooled prevalence estimate, as countries contributing more studies can exert greater weight on the regional summary. This may limit the representativeness of the pooled estimate for under-studied SSA settings where nutritional, infectious, environmental, and socio-economic conditions differ. Third, the predominance of cross-sectional studies precludes conclusions about temporality or causality. Fourth, restriction to English-language publications may have excluded relevant studies from other Sub-Saharan African settings. Finally, although pooled analyses were used to summarise broad regional patterns, these estimates should be interpreted alongside the considerable methodological and contextual variation across studies.

## 5. Conclusions

Anaemia among adolescents in SSA appears to remain an important public health concern, but the pooled estimates reported in this review should be interpreted cautiously given the substantial heterogeneity across studies and methodological differences in study populations, haemoglobin definitions, assessment methods, and analytical approaches. The findings suggest that nutritional, infectious, environmental, and socio-demographic factors may all contribute to anaemia risk in different settings; however, the available evidence does not allow firm conclusions about causality, independence of effects, or the precise magnitude of these associations across the region. These results therefore provide a broad overview of the available evidence and highlight the need for more methodologically consistent, context-specific, and longitudinal research to better inform interventions for adolescent anaemia in SSA.

## Figures and Tables

**Table 1:** Characteristics and key findings of the included studies determining the prevalence and determinants of anaemia in adolescents in Sub-Saharan Africa.

Author, Year	Country, Setting (Urban/Rural)	Study Design	Sample Size; Sex (F/M); Age Range	Anaemia/Iron Deficiency Definition	Hb Assessment Approach	Anaemia Prevalence (%)	Nutritional, Socio-Demographic, Environmental, and Infectious Determinants
Alemu et al., 2025 [22]	33 Sub-Saharan African countries	Cross-sectional	39,712; All 39,712 females; 15–19 years	Hb < 12 g/dL	Point-of-care photometric Hb; Capillary blood, HemoCue photometer	5.05%	Unimproved water supply, wealth index, rural residence, education level, water source.
Cliffer et al., 2025 [23]	Tanzania, Urban & peri-urban	Cluster-randomized controlled trial	2475; 990 females, 1485 males; 10–17 years	Hb < 12 g/dL	Point-of-care photometric Hb; Capillary blood, HemoCue Hb 201 photometer	53.3%	Stunting, sex, wealth quintile, sharing toilet facilities
Kebede et al., 2025 [24]	Ethiopia, Rural	Cross-sectional	548; All 548 females; 10–19 years	Hb < 12 g/dL	Point-of-care Hb; HemoCue Hb 201 analyser (finger prick)	25.9%	Iron–folate supplementation, Household size Deworming
Mbou et al., 2025, [25]	Cameroon, Urban	Cross-sectional	511; 259 females, 252 males; 10–15 years	Hb < 12 g/dL (girls), <13 g/dL (boys)	Point-of-care photometric Hb; Capillary blood; electronic haemoglobinometer	40.3%	Iron supplementation, illness, area residence, parental marital status, parental survival
Millogo et al., 2025 [26]	Burkina Faso, Urban, Peri-urban and rural	Cross-sectional	2947; 1621 females, 1326 males; 10–18 years	Hb < 11.5 (10–11 y), <12.0 (12–14 y/female ≥ 15 y), <13.0 (male ≥ 15 y)	Point-of-care photometric Hb; Capillary blood, HemoCue 201+ photometer	36.2%	Stunting, dietary diversity, sex, age, wealth index
Nhial et al., 2025 [27]	Ethiopia, both rural and urban	Cross-sectional study	359; All 359 females; 14–19 years	Hb < 12 g/dL	Point-of-care photometric Hb; Capillary blood, HemoCue Hb 301 photometer	28.4%	Underweight (BMI < 18.5), parasite infestation, poor dietary diversity, larger family size (≥6), poor knowledge on anaemia, lack of healthcare access, father’s life status
Partap et al., 2025 [14]	Ethiopia, Sudan, Tanzania, Urban & peri-urban	Cross-sectional	3558; 1850 females, 1708 males; 10–14 years	Hb < 11.5 g/dL (age 10–11), < 12 g/dL (age 12–14)	Point-of-care photometric Hb; Capillary blood, HemoCue analyser	32.0%	Poor diet quality, stunting, food insecurity, age, sex, school hand washing stations
Yewodiaw et al., 2025 [28]	Ethiopia, both rural and urban	Cross-sectional	345; All 345 females; 10–19 years	Hb < 12 g/dL	Point-of-care photometric Hb; Capillary blood, HemoCue Hb 301 analyser	50.4%	Poor supplementation adherence, non-vegetarian diet, drinking tea/coffee, Poor knowledge of anaemia, education level
Adokorach et al., 2024 [29]	Uganda, Rural	Cross-sectional	341; 194 females, 147 males; 10–19 years	Hb < 12 g/dL (girls), <13 g/dL (boys)	Point-of-care photometric Hb; Capillary blood, DiaSpect hand-held analyser	16.1%	No nutritional determinants found, guardian education
Osman et al., 2024 [30]	Sudan, Urban	Cross-sectional	309; 162 females. 147 males; 10 to 19 years	Hb 12 g/dL	Point-of-care photometric Hb; HemoCue Hb 301+	24.3%	Pica, no sociodemographic determinants found.
Olumakaiye, 2023 [31]	Nigeria, Rural	Cross-sectional	120; All 120 females; 10–19 years	serum ferritin < 12.0 µg/L	Ferritin assessment; Serum ferritin (blood draw),	47.5%	Low dietary diversity, low intake of both heme and non-heme iron food, age.
Yusufu et al., 2023 [32]	Tanzania, Urban & peri-urban	Cross-sectional	2479; 1517 females 962 males; 10–17 years	Hb < 11.5 (10–11 y), <12.0 (12–14 y, ≥15 y girls), <13 (≥15 y boys)	Point-of-care photometric Hb; Capillary blood, HemoCue 201 photometer	53.3%	Stunting, sex, low wealth, shared toilet facilities
Wiafe et al., 2023, [33]	Ghana, Rural	Cross-sectional	137; 67 females, 70 males; 10–14 years	Hb < 11 g/dL for both	Point-of-care photometric Hb; HemoCue Hb 301 analyser	29.9%	No nutritional determinants found, no sociodemographic determinants found.
Wemakor et al., 2023 [34]	Ghana, Rural	Cross-sectional	370; All 370 females; 10–19 years	Hb < 12 g/dL	Point-of-care photometric Hb; Capillary blood, HemoCue Hb 301 analyser	74.6%	No nutritional determinants found, guardian education; residence
Abdulai et al., 2022 [35]	Ghana, Rural	Cross-sectional	349; 138 females, 211 males; 10–19 years	Hb < 11.5 g/dL (<12 years), <12 g/dL (12–14 years), <13 g/dL (≥15 years) (boys); <11.5 or <12 g/dL for girls	Point-of-care photometric Hb; capillary blood, HemoCue Hb-301 analyser	25.2%;	Stunting, food insecurity, household wealth index
Ahmed & Mohammed, 2022 [36]	Ethiopia, Urban	Cross-sectional	372; All 372 females; 15–19 years	Hb < 12.0 g/dL;	Point-of-care photometric Hb; Capillary blood, HemoCue 301 photometer	31.5%	No nutritional determinants found, family size (>5 people), literacy
Mank et al., 2022 [37]	Burkina Faso, Urban	Cross-sectional	1059; 604 females, 455 males; 11–15 years	Hb < 11.5 g/dL (12–14 y), <12.0 (girls ≥ 15 y), <13.0 (boys ≥ 15 y)	Point-of-care photometric Hb; Capillary blood, HemoCue 201 system	50%	Thinness, gender, education level.
Annan et al., 2021 [38]	Ghana, Both rural and urban	Cross-sectional	416; All 416 females; 13–19 years	Hb < 11 g/dL	Automated haematology; Venous blood; Sysmex XP-300 haematology analyser	57.1%	Hunger, inadequate dietary diversity; mean MUAC, no sociodemographic determinants be found.
Tiruneh et al., 2021 [39]	Ethiopia	Cross-sectional	3172; All 3172 females; 15–19 years	Hb < 12.0 g/dL	Capillary blood, HemoCue analyser	23.8%	Underweight (BMI < 18.5), marital status, religion, education level
Visser et al., 2021 [40]	South Africa	Cross-sectional	578; 283 females. 295 males; 12 years	Hb < 11.5 g/dL (5–11 y); <12.0 g/dL (≥12 y)	Hb measurement; Cyanmethemoglobin method or haematology analyser	13.8%	Low dietary diversity (DDS ≤ 4), no sociodemographic determinants found.
Alemu & Gebremedhin, 2020 [41]	Ethiopia, Rural	Cross-sectional	400; All 400 females; 10–19 years	Hb < 12 g/dL,	Point-of-care photometric Hb; Capillary blood, HemoCue Hb 301 photometer	15.2%	Low dietary diversity and less frequent legume consumption, education, illiteracy, family size
Fage et al., 2020 [42]	Ethiopia, Both rural and urban	Cross-sectional	493; 172 females, 321 males	Hb < 12 g/dL (12–14 y and F ≥ 15), <13 g/dL (M15+)	Point-of-care photometric Hb; Capillary blood, HemoCue Hb 301 analyser	29.4%	Dietary diversity, sex, grades, guardian literacy, education
Fentie et al., 2020 [43]	Ethiopia, Urban	Cross-sectional	528; All 528 females; 14–19 years	Hb < 12 g/dL (females 12–19 y, males 12–14 y), Hb <13 g/dL (males 15+)	Point-of-care photometric Hb; Capillary blood, HemoCue Hb 301	26.7%	Low dietary diversity score, living separately from family, low economic status.
Gosdin et al., 2020 [44] †	Ghana, Urban	Cross-sectional	3557; 2948 females, 609 males; 10–19 years	WHO age/sex cutoffs: 10–11 y < 115 g/L, 12–14 y < 120 g/L, girls ≥ 15 y < 120 g/L, boys ≥ 15 y < 130 g/L	Point-of-care photometric Hb; Capillary blood, HemoCue 301	Girls: 24%, Boys: 13%	Geophagy, overweight, wealth gradient, knowledge of anaemia, malaria/older age
Egata et al., 2020 [45]	Ethiopia, Urban	Cross-sectional	523; All 523 females; 10–19 years	Hb <12 g/dL (≥12 y), <11.5 g/dL (10–11 y)	Point-of-care photometric Hb; Capillary blood, HemoCue HB 301 analyser	39%	Frequent tea/coffee within 30 min after meals, wasting, maternal illiteracy
Zeleke et al., 2020 [46]	Ethiopia, Both rural and urban	Cross-sectional	742; 365 females, 377 males; 10–19 years	Hb < 12 g/dL (females 12–19 y, males 12–14 y), Hb < 13 g/dL (males 15+)	Point-of-care photometric Hb; Capillary blood, HemoCue Hb 301	21.1%;	Infrequent fibre-rich food, intestinal parasite infection, age, residing in Malaria endemic area
Adelman et al., 2019 [47]	Uganda, Rural	Randomized controlled trial	233; All 233 females; 10–13 years	Hb < 11.5 g/dL (10–11 y), <12.0 g/dL (12–13 y)	Point-of-care photometric Hb; Capillary blood, HemoCue analyser	40–46%	Exposure to a Food for Education (FFE) program, no sociodemographic determinants were found.
Azupogo et al., 2019 [48]	Ghana, Rural	Cross-sectional	319; 156 females, 163 males; 10–17 years	Hb < 11.5 g/dL (6–11 y), <12 g/dL (12–14 y, females 15+), <13 g/dL (males 15+)	Point-of-care photometric Hb; Capillary blood, HemoCue Hb 201	52.3%	Household dietary diversity score, agro-ecological zone, household asset index
Gebreyesus et al., 2019 [49]	Ethiopia, Rural	Cross-sectional	1323; All 1323 females; 10–19 years	Hb < 12 g/dL	Point-of-care photometric Hb; Capillary blood, HemoCue B-Haemoglobin analyser	29.2%	Food insecurity, lack of anaemia awareness, age, rural residence
Mengistu et al., 2019 [50]	Ethiopia, Rural	Cross-sectional	423; All 423 females; 10–19 years	Hb < 12 g/dL (mild)	Point-of-care photometric Hb; Capillary blood, HemoCue photometer	11.1%	History of intestinal parasitic infection, thinness, low family income (<500 ETB), family size > 5
Regasa & Haidar, 2019 [51]	Ethiopia, Rural	Cross-sectional	448; All 448 females; 10–19 years	Hb < 12 g/dL	Point-of-care photometric Hb; Capillary blood, HemoCue 301 photometer	27%	Nutritional determinants not found, age, rural residence.
Engidaw et al., 2018 [52]	Ethiopia, Both rural and urban	Cross-sectional	437; All 437 females; 10–19 years	Hb < 12.5 g/dL	Point-of-care photometric Hb; Capillary blood, HemoCue Hb 301	22%	Low intake of heme iron-rich foods (<1 time/month), low egg consumption, age
Gonete et al., 2018 [53]	Ethiopia, Both rural and urban	Cross-sectional	462; All 462 females; 15–19 years	Hb < 12 g/dL	Point-of-care photometric Hb; Capillary blood, Hb201 photometer	25.5%	Inadequate dietary diversity, household food insecurity, living with either parent or guardians compared to both parents.
Shaka & Wondimagegne, 2018 [54]	Ethiopia, Both rural and urban	Cross-sectional	424; 177 females, 247 males; 10–19 years	Hb < 11.5 g/dL (≤11 y), <12 g/dL (12–14 y + F ≥ 15 y), <13 g/dL (M ≥ 15 y)	Point-of-care photometric Hb; Capillary blood, e HemoCue meter	22%	Stunting, low meal frequency, large family size (>5 family members); source of food (purchase), rural residence, rarely wearing shoes, family wealth, sex.
Ayogu et al., 2016 [55]	Nigeria, Urban	Cross-sectional	400; 175 females, 225 males; 12–18 years	Hb < 12 g/dL	Hb measurement; Venous blood; cyanmethemoglobin method	64.0%	Low dietary intake of iron-rich foods, high prevalence of stunting and thinness, household income
Intiful et al., 2016 [56]	Ghana, Urban	Cross-sectional	265; All 265 females; 15–19 years	Hb < 11 g/dL	Hb measurement; Venous blood, automated full blood count	76%	Pica practice, no sociodemographic determinants were found.
Teji et al., 2016 [57]	Ethiopia, Both rural and urban	Cross-sectional	547; All 547 females; 10–19 years	Hb < 12 g/dL	Point-of-care photometric Hb; Capillary blood, HemoCue portable photometer	32.0%	Stunting, rural residence, older adolescent age (15–19 years), farmer father occupation, non-piped water.
Adem, 2015 [58]	Ethiopia, both rural and urban	Cross-sectional	338; All 338 females; 14–19 years	Hb < 12 g/dL	Capillary blood, HemoCue Hb 301 analyser	22.8%	Low frequency consumption of meat, eggs, vegetables and fruits, and high milk intake, low socioeconomic status
Sasie & Murutse, 2015 [59]	Ethiopia, Urban	Cross-sectional	570; All 570 females; 15–19 years	Hb < 12 g/dL	Point-of-care photometric Hb; Capillary blood, HemoCue 301 analyser	21.1%	Low consumption of animal products, low vegetable intake, tea/coffee after meals, and being underweight, low maternal education.
Nelima, 2015 [60]	Kenya, Both rural and urban	Cross-sectional	230; All 230 females; 14–18 years	Hb < 12.0 g/dL	Point-of-care photometric Hb; Venous blood, HemoCue Hb 201 Analyzer	26.5%	Inadequate iron intake, malaria, worm infestation, age, low paternal education.
Tesfaye et al., 2015 [61]	Ethiopia, Urban	Cross-sectional	408; 221 females, 187 males; 12–19 years	Hb < 12 g/dL (females ≥ 12 y, males 12–14 y), <13 g/dL (males > 15 y)	Automated haematology; Venous blood (CELL-DYN 1800), Abbott Lab	15.2%	Dietary intake, sex, place of residence,
Assefa et al., 2014 [62]	Ethiopia, Urban	Cross-sectional	404; 218 females, 186 males; 6–14 years	Hb < 12 g/dL (12–14 y); <11.5 g/dL (6–11 y)	Point-of-care photometric Hb; Capillary blood, HemoCue digital photometer	37.6%;	Infrequent intake of plant and animal foods, low family income, low maternal education.
Orish et al., 2012 [63]	Ghana, Rural	Cross-sectional	866; All 866 females; 15–19 years	Hb < 11 g/dL	Point-of-care photometric Hb; Venous blood, cyanmethemoglobin method	43.9%	No dietary determinants were found, adolescent pregnancy.
Abdelrahim, 2009 [64]	Sudan, Both rural and urban	Cross-sectional	187; All 187 females; 11–18 years	Hb < 12 g/dL	Point-of-care photometric Hb; Capillary blood, HemoCue haemoglobinometer	96.8%	Micronutrient deficiencies, no sociodemographic determinants were found.
Alaofe et al., 2008 [65]	Benin, Urban	Cross-sectional	180; All 180 females; 12–17 years	Hb < 12 g/dL	Automated haematology; Venous blood, Sysmex KX-21 haematology analyser	51%	Low meat consumption (<4/week); low fruit (<4/week), no sociodemographic determinants were found.
Woodruff et al., 2006 [66]	Kenya & Nepal, Refugee camps	Cross-sectional	Kenya: 391, Nepal 462; Kenya: 262 males; 129 females. Nepal: 336 females,126 males; 10–19 years	Hb < 11.5 (10–11 y), <12.0 (12–14 y/girls ≥ 15 y), <13.0 (boys ≥ 15 y)	Point-of-care photometric Hb;Capillary blood, HemoCue haemoglobinometer	Kenya: 45.9% Nepal: 23.8%	High iron deficiency, age.
Leenstra et al., 2004 [67] †	Kenya, Rural	Cross-sectional	648; All 648 females; 12–18 years	Hb < 120 g/L	Hb measurement; Fingerpick blood, Coulter Counter (lab); HemoCue	21.1%	No nutritional determinants were found, age.
Massawe et al., 2003 [68] †	Tanzania, Urban	Cross-sectional	231; 130 females, 101 males; 12–19 years	Hb < 120 g/L for adolescents; Hb < 105 g/L for pregnant girls;	Automated haematology; Venous blood; ABX Micros CBS 18 Coulter haematological analyser	14.6%	Hookworm infection, sex.

Note: † Gosdin et al. (2020), Leenstra et al. (2004), and Massawe et al. (2003) reported haemoglobin thresholds in g/L rather than g/dL as used by all other studies. These values are consistent with WHO-recommended cut-offs when expressed in equivalent units (1 g/dL = 10 g/L). Hb: haemoglobin [44,67,68]. Point-of-care haemoglobin device manufacturers: HemoCue (HemoCue AB, Ängelholm, Sweden); DiaSpect (EKF Diagnostics, Cardiff, UK); Sysmex XP-300 (Sysmex Corporation, Kobe, Japan); ABX Micros CBS 18/Coulter-type analyzers (HORIBA ABX SAS, Montpellier, France).

**Table 2:** Multivariate meta-regression analysis of anaemia prevalence among adolescents in SSA.

Variables	Categories	Multivariate Meta Regression		
		Coefficient	*p*-Value	*I* ^2^
Sample size		−0.0001	0.0080 **	99.26
Publication Year		−0.0048	0.8681	99.26
Study Setting	Urban	−0.1475	0.6719	99.26
	Rural	−0.4228	0.2106	99.26
	Mixed	−0.4648	0.3751	99.26
	Refugee Camp	−0.0834	0.9140	99.26
Hb assessment	HemoCue (Point-of-Care)	−0.2120	0.6665	99.26
	Cyanmethemoglobin Method	0.1547	0.8200	99.26
	Automated Haematology Analyzers	−0.4030	0.6248	99.26
	Unclear/Other	−0.0711	0.9271	99.26

** = *p* value < 0.01.

**Figure A1:**
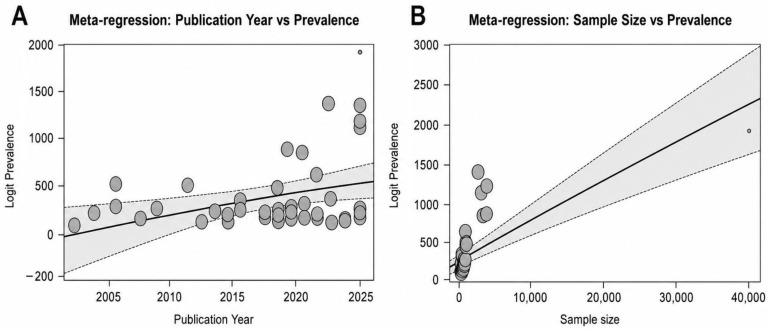
Meta-regression bubble plots examining sources of heterogeneity in the prevalence of anaemia among adolescents in SSA. (**A**) Publication year and (**B**) Total sample size. The solid line represents the fitted meta-regression line, and the shaded area with dashed lines indicates the 95% confidence interval.

## Data Availability

No new data were created or generated in this systematic review. All data analyzed in this study were obtained from previously published studies, which are cited in the article and listed in the References Section. Data extracted and synthesized for the review are available from the corresponding author upon reasonable request.

## Supplementary Materials

The following supporting information can be downloaded at https://www.mdpi.com/article/10.3390/nu18152404/s1. Supplementary File S1: The full search strategy. Supplementary File S2: JBI appraisal results for all included studies (Table S1).

## Appendix A. Meta Regression Analysis of Association

**Table A1:** Factors related to the heterogeneity of prevalence of anaemia in the current meta-analysis (multi-meta-regression model).

Variables	Coefficient	*p*-Value	Standard Error
**Socio-demographics**			
**Publication Year**	0.0075	0.2048	0.0366
**Sample Size**	−0.0004	0.0020 *	0.0001
**Dietary diversity**			
**Publication Year**	0.0505	0.5719	0.0893
**Sample Size**	−0.0004	0.0406 *	0.0002
**Anthropometric undernutrition**			
**Publication Year**	−0.0126	0.8264	0.0575
**Sample Size**	−0.0003	0.0383 *	0.0002
**Infection-related nutritional factors**			
**Publication Year**	−0.0357	0.3837	0.0410
**Sample Size**	−0.0004	0.0237 *	0.0002
**Environmental determinants**			
**Publication Year**	0.2110	<0.0001 **	0.0485
**Sample Size**	−0.0007	<0.0001 **	0.0002

* = *p* value < 0.05, ** = 0.0001.
