# Supplementary material for: Prevalence and Factors Associated with Anaemia Among Sub-Saharan African Adolescents: A Systematic Review and Meta-Analysis of Nutritional, Socio-Demographic, Environmental, and Infectious Factors
Source: Nutrients. 2026 Jul 23;18(15):2404. doi: 10.3390/nu18152404 (PMC13467683; doi:10.3390/nu18152404)
Supplement: Supplementary file 1 [file nutrients-18-02404-s001.zip › nutrients-4375565-supplementary.pdf]

Supplementary File 1 : The full search strategy for the systematic review

(adolescent*[tiab] OR teen*[tiab] OR youth[tiab] OR “secondary school”[tiab]) AND (anemi*[tiab] OR anaemi*[tiab] OR “iron deficiency”[tiab] OR haemoglobin[tiab] OR hemoglobin[tiab]) AND (“Africa South of the Sahara”[MeSH Terms] OR (Angola[tiab] OR Benin[tiab] OR Botswana[tiab] OR “Burkina Faso"[tiab] OR Burundi[tiab] OR Cameroon[tiab] OR “Cape Verde”[tiab] OR “Central African Republic”[tiab] OR Chad[tiab] OR Comoros[tiab] OR Congo[tiab] OR "Democratic Republic of Congo”[tiab] OR “Côte d’Ivoire”[tiab] OR Djibouti[tiab] OR Eritrea[tiab] OR Eswatini[tiab] OR Ethiopia[tiab] OR Gabon[tiab] OR Gambia[tiab] OR Ghana[tiab] OR Guinea[tiab] OR “Guinea-Bissau”[tiab] OR Kenya[tiab] OR Lesotho[tiab] OR Liberia[tiab] OR Madagascar[tiab] OR Malawi[tiab] OR Mali[tiab] OR Mauritania[tiab] OR Mozambique[tiab] OR Namibia[tiab] OR Niger[tiab] OR Nigeria[tiab] OR Rwanda[tiab] OR “Sao Tome”[tiab] OR Senegal[tiab] OR Seychelles[tiab] OR “Sierra Leone”[tiab] OR Somalia[tiab] OR “South Africa”[tiab] OR “South Sudan”[tiab] OR Sudan[tiab] OR Tanzania[tiab] OR Togo[tiab] OR Uganda[tiab] OR Zambia[tiab] OR Zimbabwe[tiab]))

Prevalence and determinants of anaemia in adolescents (10–19 y) in Sub-Saharan Africa: a systematic review of nutritional and socio-demographic risk factors
Google scholar "Anaemia in adolescents” AND (“Sub-Saharan Africa” OR Africa) AND (“nutritional risk factors” OR “socio-demographic”) Apply filter 2000 to 2025
“Anaemia in adolescents” AND (“Sub-Saharan Africa” OR Africa) AND (“nutritional risk factors” OR “socio-demographic”)

(adolescen* OR teen* OR youth OR “secondary school”) AND (anemi* OR anemia OR “iron deficiency” OR haemoglobin OR hemoglobin) AND (“Sub-Saharan Africa” OR “Africa South of the Sahara” OR Angola OR Benin OR Botswana OR “Burkina Faso” OR Burundi OR Cameroon OR “Cape Verde” OR “Central African Republic” OR Chad OR Comoros OR Congo OR “Democratic Republic of Congo” OR “Côte d'Ivoire” OR Djibouti OR Eritrea OR Eswatini OR Ethiopia OR Gabon OR Gambia OR Ghana OR Guinea OR “Guinea-Bissau” OR Kenya OR Lesotho OR Liberia OR Madagascar OR Malawi OR Mali OR Mauritania OR Mozambique OR Namibia OR Niger OR Nigeria OR Rwanda OR “Sao Tome” OR Senegal OR Seychelles OR “Sierra Leone” OR Somalia OR “South Africa” OR “South Sudan” OR Sudan OR Tanzania OR Togo OR Uganda OR Zambia OR Zimbabwe) AND (nutrition* OR “nutritional status” OR diet* OR malnutrition OR micronutrient OR iron OR “socio-demographic” OR socioeconomic OR education OR income OR “risk factor*”)

Supplementary file 2 : JBI appraisal results for all included studies (Table S1)

**Author, Year**	**q1**	**q2**	**q3**	**q4**	**q5**	**q6**
Alemu et al., 2025	Yes	Yes	Yes	Yes	Yes	Yes
Cliffer et al., 2025	Yes	Yes	Yes	Yes	Yes	Yes
Kebede et al., 2025	Yes	Yes	Yes	Yes	Yes	Yes
Mbou et al., 2025	Yes	Unclear	Yes	Yes	Unclear	Yes
Millogo et al., 2025	Yes	Yes	Yes	Yes	Yes	Yes
Nhial et al., 2025	Yes	Yes	Yes	Yes	Yes	Yes
Partap et al., 2025	Yes	Yes	Yes	Yes	Yes	Yes
Yewodiaw et al., 2025	Yes	Yes	Yes	Yes	Yes	Yes
Adokorach et al., 2024	Yes	Yes	Yes	Yes	Yes	Yes
Assefa et al., 2024	Yes	Yes	Yes	Yes	Yes	Yes
Osman et al., 2024	Yes	Yes	Yes	Yes	Yes	Yes
Olumakaiye, 2023	Yes	Unclear	Unclear	Yes	Unclear	Unclear
Yusufu et al., 2023	Yes	Yes	Unclear	Yes	Yes	Yes
Wiafe et al., 2023	Yes	Unclear	Yes	Yes	Unclear	Yes
Wemakor et al., 2023	Yes	Yes	Yes	Yes	Yes	Yes
Abdulai et al., 2022	Yes	Yes	Yes	Yes	Yes	Yes
Ahmed & Mohammed, 2022	Yes	Yes	Yes	Yes	Yes	Yes
Mank et al., 2022	Yes	Unclear	Yes	Yes	Unclear	Yes
Annan et al., 2021	Yes	Unclear	Yes	Yes	Yes	Yes
Tiruneh et al., 2021	Yes	Yes	Yes	Yes	Yes	Unclear
Visser et al., 2021	No	No	Unclear	Yes	Unclear	Yes
Alemu & Gebremedhin, 2020	Yes	Yes	Yes	Yes	Yes	Unclear
Fage et al., 2020	Yes	Yes	Yes	Yes	Yes	Yes
Fentie et al., 2020	Yes	Yes	Yes	Yes	Yes	Yes
Gosdin et al., 2020	Yes	Yes	Yes	Yes	Yes	Yes
Tura et al., 2020	Yes	Yes	Yes	Yes	Yes	Yes
Zeleke et al., 2020	Yes	Yes	Yes	Yes	Yes	Yes
Adelman et al., 2019	Yes	Yes	Yes	Yes	Yes	Yes
Azupogo et al., 2019	Yes	Yes	Yes	Yes	Yes	Yes
Gebreyesus et al., 2019	Yes	Yes	Yes	Yes	Yes	Yes
Mengistu et al., 2019	Yes	Yes	Yes	Yes	Yes	Yes
Regasa & Haidar, 2019	Yes	Yes	Yes	Yes	Yes	Yes
Engidaw et al., 2018	Yes	Yes	Yes	Yes	Yes	Yes
Gonete et al., 2018	Yes	Yes	Yes	Yes	Yes	Yes
Shaka & Wondimagegne, 2018	Yes	Yes	Yes	Yes	Yes	Yes
Ayogu et al., 2016	Yes	Yes	No	Yes	Unclear	Yes
Intiful et al., 2016	Yes	Unclear	Unclear	Yes	Yes	Yes
Teji et al., 2016	Yes	Yes	Yes	Yes	Yes	Yes
Adem, 2015	Yes	Yes	Yes	Yes	Yes	Unclear
Damelash & Murutse, 2015	Yes	Yes	Yes	Yes	Yes	Yes
Nelima, 2015 [56]	Yes	Yes	Yes	Yes	Yes	Yes
Tesfaye et al., 2015	Yes	Yes	Yes	Yes	Yes	Yes

Orish et al., 2012	Unclear	Unclear	Unclear	Yes	Unclear	Yes
Abdelrahim, 2009	Yes	Yes	Unclear	Yes	Unclear	Yes
Alaofè et al., 2008	Yes	Yes	Unclear	Yes	Unclear	Yes
Woodruff et al., 2006	Yes	Yes	Yes	Yes	Yes	Yes
Leenstra et al., 2004	Yes	Yes	Yes	Yes	Yes	Yes
Massawe et al., 2002	Unclear	Unclear	Unclear	Yes	Unclear	Yes

Yes=1
No=0
Unclear=0
N/A=0

JBI Risk of Bias and Quality Assessment Summary

**Total studies**	48

**Overall appraisal**	**Count**
Include	33
Include with caution	15
Exclude	0

**Criterion**	**Yes**	**Unclear**	**No**	**NA**
q1	45	2	1	0
q2	39	8	1	0
q3	39	8	1	0
q4	48	0	0	0
q5	38	10	0	0
q6	44	4	0	0
q7	45	3	0	0
q8	47	1	0	0
q9	29	16	0	3

**Criterion**	**Description**
q1	Was the sample frame appropriate to address the target population?
q2	Were study participants sampled in an appropriate way?
q3	Was the sample size adequate?
q4	Were the study subjects and setting described in detail?
q5	Was data analysis conducted with sufficient coverage of the identified sample?
q6	Were valid methods used for identification of the condition?
q7	Was the condition measured in a standard, reliable way for all participants?
q8	Was there appropriate statistical analysis?
q9	Was the response rate adequate, and if not, was low response managed appropriately?
